# Usability Pitfalls of Diabetes mHealth Apps for the Elderly

**DOI:** 10.1155/2016/1604609

**Published:** 2016-02-29

**Authors:** Maša Isaković, Urban Sedlar, Mojca Volk, Janez Bešter

**Affiliations:** Faculty of Electrical Engineering, University of Ljubljana, Tržaška Cesta 25, SI-1000, Ljubljana, Slovenia

## Abstract

Diabetes mellitus has high prevalence in the ageing population and is often accompanied by other comorbidities, such as Alzheimer's disease, and general disabilities, such as poor eyesight. These comorbidities have redefined ways in which patients use mHealth apps, including diabetes apps. The latter have proven benefits for monitoring blood glucose levels and insulin tracking in the general population. In this paper, we analyse a diabetes monitoring app DeStress Assistant (DeSA), which was developed as a part of an EU project and tested in a hospital setting. Due to the increasing number of older adults, we wanted to ensure the app was suitable for that demographic. Based on a number of supervised tests, we show that the app, which was developed with the help of workshops and feedback from tech-savvy patients and clinicians, is difficult to use by elderly users. We demonstrate that with a small number of changes it is possible to raise the usability of the app in a number of categories. We summarise the lessons learned in the discussion. Our findings demonstrate that special care needs to be taken when developing mHealth apps for the elderly population.

## 1. Introduction

Age is associated with changes in sensory abilities; the most-studied age-associated sensory changes are vision and audition [1]. Colour vision, contrast sensitivity, and visual acuity all decline with age. Also, aspects of memory (e.g., keeping a lot of information active in working memory), online reasoning ability, and aspects of attention, such as attending to more than one source of information all show age-related declines [2].

With the increase in numbers of the aging population, we can also see an increase of chronic diseases such as diabetes mellitus. Type II diabetes affects 90% of people with diabetes around the world and is largely the result of excess body weight and physical inactivity [3]. Self-monitoring blood glucose (SMBG) systems have the potential to play an important role in the management of diabetes and in the reduction of risk of serious secondary clinical complications [4]. In that regard, mobile applications can be used as an effective tool in different self-monitoring techniques. They are useful for all user groups from people with no overt health problems to chronic patients. They have been tested on patients with type II diabetes, asthma, chronic obstructive pulmonary disease (COPD), and different psychiatric conditions [5–8].

In this paper, we investigate on a practical example how elderly people specifically use mobile applications for diabetes management. We describe the limitations that might be preventing them from adopting such digital mHealth solutions and outline and demonstrate how existing applications can be adapted to increase the usability and adoption rate.

Our findings will also be put into practice with our involvement in the UNCAP [9] project, which is aimed at adapting digital health solutions and technologies for the aging population, thus helping them live independently while maintaining and improving their lifestyle.

The remainder of this paper is organized as follows.  Section 2 presents the DeSA app and all its functionalities; Section 3 introduces the utilized methodology, where the moderating technique and the questionnaire are discussed.  Section 4 combines the results from the individual evaluations and demonstrates the changes made to the app.  Section 5 presents our discussion, and Section 6 concludes the paper.

### 1.1. The Rise of Diabetes Prevalence

One of the most important demographic changes to diabetes prevalence across the world appears to be the increase in the proportion of people over 65 years of age. Furthermore, the number of elderly persons with type II diabetes is expected to grow in concert with the overall increase of the elderly population. The majority of people with diabetes in developed countries are older than 64 years. By 2030, it is estimated that the number of people with diabetes over the age of 64 will be more than 82 million in developing countries and more than 48 million in developed countries [11].

### 1.2. mHealth Apps

Mobile phones are becoming an increasingly important tool in the areas of health protection. The benefits include an increased feeling of safety, time and cost savings, shortened waiting queues, improved quality of life, and possibilities to develop additional health-related activities [12]. mHealth applications use mobile devices for collecting healthcare data, delivering healthcare data to physicians, researchers and patients, monitoring vital signs in real time and ensuring direct healthcare (i.e., telemedicine). Examples include the exchange of medical information via email, texting, smartphone apps, storing and forwarding pictures, and web-based video [13].

According to the IMS Institute for Healthcare Informatics [14], in the last years, the number of mobile health apps has soared with more than 165,000 mHealth apps available in the Apple App Store and Google Play. The number of apps in the Apple App Store has doubled since 2013, with more than 90,000 available apps. However, only 10 percent of mHealth apps can connect to a device or sensor that provides access to medical data.

There are more than 1,700 diabetes apps in all the app stores combined (Google play, Apple App Store, Blackberry, Windows, and Ovi Store) [15]. Researchers have examined outcomes of interventions using some of these mobile phone apps for diabetes. In [6], where studies of the clinical effectiveness of mobile-based applications were reviewed, they found 10 of the 13 studies in type II diabetes and 4 of the 7 studies on type I diabetes found mHealth to lead to benefits. Other studies [16, 17] have also found promise in using mobile app interventions.

### 1.3. Adoption of Apps

Considering the number of apps, the users are left to their own devices to find an app that they feel helps them manage their medical condition in the most beneficial way. After the app is developed and available on the market, the developers normally receive very little feedback and have no clear understanding as to what the consumers find engaging and useful, which can significantly undermine the potential effectiveness of the intervention. Regrettably, there is no simple formula for designing engaging and effective mHealth apps [18], and the matter has to be addressed on a case-by-case basis.

As opposed to fitness and well-being apps, mHealth technologies are frequently designed and developed within the scope of the existing structures of the health care system. However, when including patients as part of the design team, out-of-the-box thinking is encouraged, inspiring that designers or care providers who develop the technologies to think differently, unconventionally, or from a new perspective, finally leading to applications that are better tailored to patients' needs [19]. However, during such processes, many aspects can be overlooked. For example, the technology developers often fail to fully capture the tacit knowledge and develop useless solutions that do not address the real problems. Similarly, many other possible stakeholder groups can be overlooked, including the elderly. This is becoming unacceptable in an increasing number of fields, including diabetes, where the target market of the solution significantly overlaps with the elderly demographic group. If the capabilities and limitations of the elderly group are not considered in the design and development process of mHealth applications, most likely results will be poor adoption and inefficient use of the technology, thus negating all resources spent on trying to solve the problem.

## 2. The DeStress Assistant (DeSA)

The goal of the study was to ascertain whether an application that was designed for general population can be efficiently used by the elderly without modifications. In our tests we used an in-house developed diabetes application called the DeStress Assistant (DeSA) [20]. After the first round of tests, several modifications were made to the application, and the tests were repeated with a different set of users.

DeSA was designed and developed within the FISTAR project [21], with the goal of providing diabetes patients that live in remote areas a way to track their condition and keep in touch with their physician. During the design phase, no special consideration was given to the elderly demographic. Medical staff, diabetes patients, and their next-of-kin were involved during the design phase to ensure the best possible user experience and compliance with clinically related requirements. After the initial app was developed and tested in a hospital setting, with a group of patients of mixed demographic structure, we tested the app again, in a nonclinical setting, with a group of elderly users. This led to identification of the possible design flaws impeding the adoption of the application within the older demographic.

Since DeSA was developed based on feedback from multiple stakeholder groups (patients, next-of-kin, clinicians, medical device makers, developers, and information security specialists), it contains a broad spectrum of features. These include glucose diary, automatic logging of physical activity using the in-built motion sensors or add-on pedometers, macronutrient logging, self-reporting of stress, and weight and insulin boluses, as well as data logging reminders and sharing of logs directly with a physician.

DeSA is designed as a self-contained application that stores all the observation data on the phone. During the design phase, to comply with privacy regulations, special care was taken to ensure that data never leaves the device without the user's consent [23].

**Figure 1:**
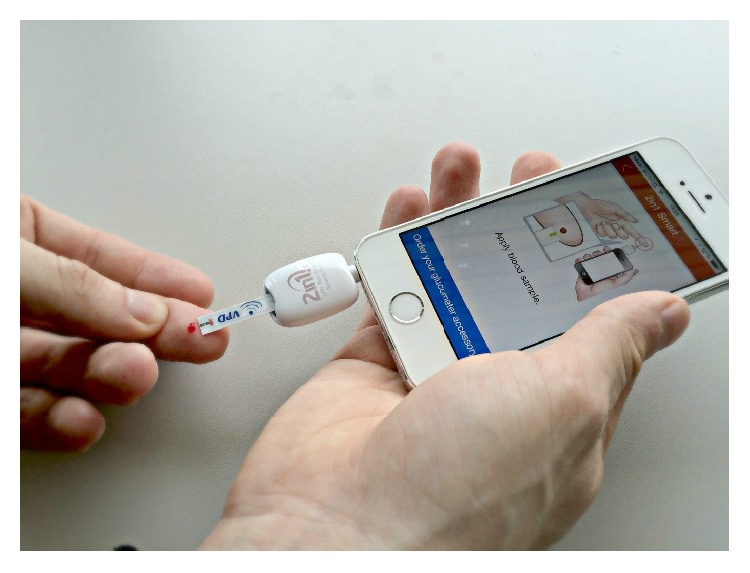
Performing the blood glucose measurement.

In a systematic review [22] of diabetes applications, only 17.7% of apps on the market at that time offered three or more functions and only a small number of apps offered the possibility of a connected glucometer device and transferring of data wirelessly and automatically via Bluetooth to the mobile device. DeSA was specifically designed to interface with the glucometer directly, by plugging the glucometer directly into the audio jack of the mobile device. This eliminated several possible issues, including charging the batteries of the glucometer, and provided greater security and reliability than transferring data radio-based technologies such as Bluetooth (see Figure 1).

## 3. Usability Evaluation Methodology

It is recommended that applications be developed with the end user in mind [18]. This proves crucial if the application aims to be both useful and usable. With DeSA, multiple stakeholders were involved in the design and development process, where their needs, wants, and limitations were given special attention at each stage. With this study, we aimed to repeat the usability evaluation on an older population. To do so, the usability evaluation method needed to be adapted with the end user in mind.

The best way to ensure usability is to have the potential users (as well as other stakeholders) involved in the process of solution design and development. That way, the developers can understand the needs of the users and can address potential issues before the app is finished. However, once the app has been developed, and a lot of research and development resources went into it, changes become increasingly hard. The optimization problem that we wanted to address was how to improve the app without going through the whole process again and achieve maximum improvement with minimum effective modification. For this it was important to first identify and understand the most pressing issues the users face and based on the findings and modify the app accordingly.

The first step of the evaluation process is determining whether the app should be evaluated by the experts or by the end users. Based on the extensive previous evaluations performed both by general population and by domain experts, we have chosen to perform subsequent usability evaluations on end users alone. The users were all older than 50, with the average age of 64.2 years. Some had experience with touchscreen technology, while others did not. The first step of the evaluation was thus choosing a moderating technique and designing a suitable questionnaire, as follows.

### 3.1. Moderating Techniques

Effectively moderating usability tests plays a crucial part in understanding the needs of the users. The most common moderation techniques include the following [24]:

- Concurrent Think Aloud (CTA) is used to understand participants' thoughts as they interact with a product by having them think aloud while they work. The goal is to encourage participants to keep a running stream of consciousness as they work.
- In Retrospective Think Aloud (RTA), the moderator asks participants to retrace their steps when the session is complete. Often participants watch a video replay of their actions, which may or may not contain eye-gaze patterns.
- *Concurrent Probing* (CP) requires that as participants work on tasks—when they say something interesting or do something unique, the researcher asks follow-up questions.
- Retrospective Probing (RP) requires waiting until the session is complete and then asking questions about the participant's thoughts and actions. Researchers often use RP in conjunction with other methods—as the participant makes comments or actions, the researcher takes notes and follows up with additional questions at the end of the session.

Our usability tests were performed in two phases on *N* = 10 users. The used moderating technique was the RP with the combination of CTA, which seemed to fit the target audience best. We decided not to use the CP technique, because we wanted to let the user navigate the app freely and not lose focus. Considering the age group, we also decided the RTA would increase the overall length of the session and likely cause them to lose focus. The elderly users were asked to perform a set of tasks in the application, while the moderator observed and provided limited assistance, if needed. After the participants completed the tasks, they were asked a set of questions, to determine how they felt using the app and to try and distinguish the possible difficulties they encountered while using the app.

### 3.2. Questionnaire

There is a wide variety of questionnaires available for testing usability and user experience. The questionnaire has to be designed specifically for the end users, and not the experts evaluating the application in question; even more importantly, it has to yield specific results. Questionnaires designed for experts typically involve testing by individuals familiar with technology and experience with using different mobile apps. For the improvement and adaptation of the app, more than just a degree of satisfaction is needed. We need to highlight specific issues that have to be handled in order for the app to be beneficial for the older demographic.

In the scientific literature, we encountered various tools used to assess the quality of mobile applications. One such tool is the Mobile App Rating Scale (MARS), developed by [25]. They considered existing guidelines for evaluating the usability of mHealth apps and came to the conclusion that they were incomplete and a reliable and objective instrument was still needed. For this reason, they developed a multidimensional scale for classifying and rating the quality of mobile health apps.

The largest drawback of the MARS scale, with respect to the elderly, is the fact that it recommends the evaluators complete a training exercise and thoroughly explore the app's content and functionalities. This means that users that are not familiar with the app will not be able to fully rate it. The questionnaire is also far too complex for the average user and it is not geared towards elderly users. In fact, the main reason for the training exercise is the complexity of the questionnaire.

After careful consideration, we decided to adapt the questionnaire developed by [22] instead of the MARS scale. Due to the complexity of most questionnaires and their target audience not being elderly users, we decided to use one that would allow us to gather as much useful information as possible and at the same time not confuse and strain the respondents. The questionnaire was not primarily developed for elderly users to evaluate the app but was adapted and yielded quick and specific results. However, some questions still had to be answered by an expert, because they were either beyond the scope of the performed test, or because they were based on experiencing rare occurrences. For example, the questions about fault tolerance can only be answered, if a mistake is made while entering data. Since the users in our case have not encountered that issue and could not know how or if the app manages erroneous input, they could not answer this question.

In addition to the chosen and adapted questionnaire (see Table 1), we also decided to use the System Usability Scale (SUS), as SUS has become an industry standard, with references in over 1300 articles and publications [26]. SUS was not intended to diagnose usability problems, but it can be used for benchmarking outside of a usability test. In our care, it would serve as a comparison of perceived usability between before and after the applied changes.

SUS provides a reliable tool for scoring the usability. It consists of a 10-item questionnaire with 5 response options for respondents, from “Strongly agree” to “Strongly disagree.”

**Figure 2:**
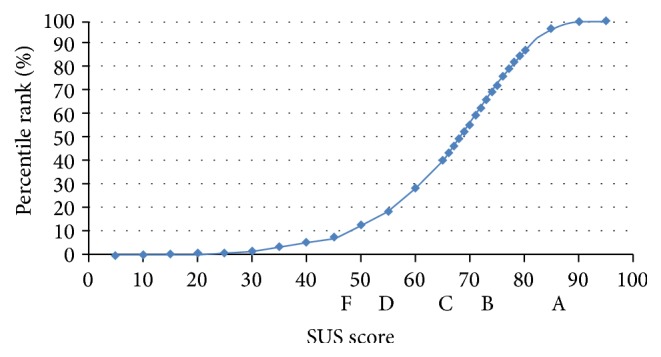
Association between percentile ranks, SUS scores, and letter grades [10].

In order to get the best ranking (A), a score above an 80.3 is needed (see Figure 2). This is also the point where users are more likely to be recommending the product to a friend. Scoring at the mean score of 68 equals a C and anything below a 51 is an F (the bottom 15%).

Age-specific requirements, such as screen size, colour, and use of symbols normally familiar to younger users, can cause usability issues in older users. Also, differentiations between clickable and unclickable areas, all play an important role in the end user's desire to use the application. Our objective was to determine specific issues and relay that information to the developers as simply and clearly as possible, so the problems could be dealt with and the app could be optimised for older users. The results can also be used as a guide when designing applications for the elderly, as they offer certain guidelines and highlight important areas.

## 4. Results

**Figure 3:**
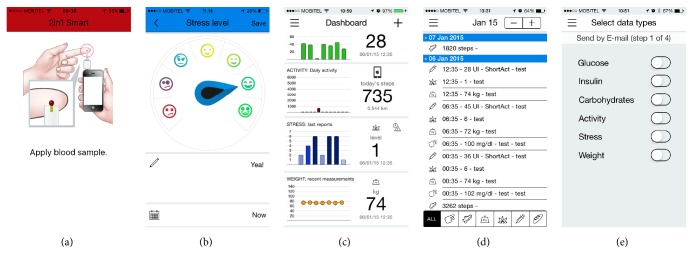
(a) Measuring glucose with the device, (b) assessing stress level, (c) chart overview, (d) logbook, and (e) sending observations by email.

The first test included users over the age of fifty, with the average age of 64. They were instructed to open the application and perform the following tasks: (1) measure their blood glucose level, (2) record their stress level, (3) view the data on the charts, (4) review the data in the logbook, and (5) send the data to their physician (see Figure 3). The moderator was observing the user and helping them with simple suggestions, if they did not know how to perform the task. After the test was completed, they were asked to answer the two chosen questionnaires and grade their experience.

The questions that were deemed unsuitable for the users, specifically the criterion of “general characteristics,” were answered by an expert. This was done because we consider the fault tolerance/fault management to be an important aspect of mHealth apps and should be handled appropriately. If a user does not input an erroneous value during the test, they would never notice this feature was not available, but a regular user would encounter this problem eventually.

Analysing the results, the evaluations were in the range of 3.0 to 4.3, which corresponds to a moderate to good rating of the app. The app received the lowest rating for the criterion “comprehensibility,” specifically the subcriterion of “simple comprehensibility and interpretability of displayed images and depictions,” with a total average value of 2.6 (the rationale for the low score by the users was “The symbols do not look like buttons. They look like random images.”, “I could not find the ‘add' and ‘menu' buttons”, etc.).

The lowest scored characteristic was the “provision of additional explanations,” with the average value of 1.8. This rating comes as no surprise, as there was no welcome wizard or any other help in the app. The app received the best rating for the criterion “presentation” (4.3) followed by “usability” (3.5) (the rationale for the good rating in “presentation” was “The screen size is very good. I can use the application without my glasses”, “I like the colours used in the app and the text is big enough”, etc.).

The criterion valued by the expert was rated the worst with the value of 1, due to the fact that the subcriterion of “high fault tolerance/efficient fault management” was not dealt with properly in the app. The data input was not limited to meaningful values and there was a lack of user feedback in the case of erroneous data input. Both of these problems were also detected by one of the users who had issues measuring blood glucose automatically and decided to input it manually. When they made a mistake while choosing the blood glucose level, they were not alerted to a possible error.

The following categories of problems were represented the most:

- The app was missing a welcome wizard with instructions to help the user get started.
- The icons used on buttons were confusing; there were problems differentiating between a button and an image or text.
- The app was missing error notifications (e.g., in case of erroneous input).
- The input data was not validated or limited to meaningful values (e.g., the possibility of choosing dates in the future).

The users found the first screen (dashboard) of the newly installed app confusing, due to the lack of any data or instructions. There were problems distinguishing the buttons on the screen; only the users with prior smartphone experience could find the add (+) button, but even these could not find the menu (≡) button (see Figure 5(a)). All the other users had to be aided in finding both buttons to initiate the testing procedure.

If the welcome wizard would guide the user and offer an explanation of the different button symbols and what they represent, the user might find the application easier to use. The two most common cognitive declines that accompany aging both affect the memory function, either the working memory, that is, the ability to maintain information actively as it is being processed, or the episodic memory, which has the ability to store new memories of events [27]. Therefore, it might be more beneficial to make the buttons clearer and simplify the use. This eliminates the need to read instructions every time the app is used. The buttons should have text or symbols with text, so the user knows what they represent.

The SUS score of the first test was 64.4 (see Figure 6(b)), which gives it a D grade or the percentile rank of 30%.

### 4.1. Improving the Usability

**Figure 4:**
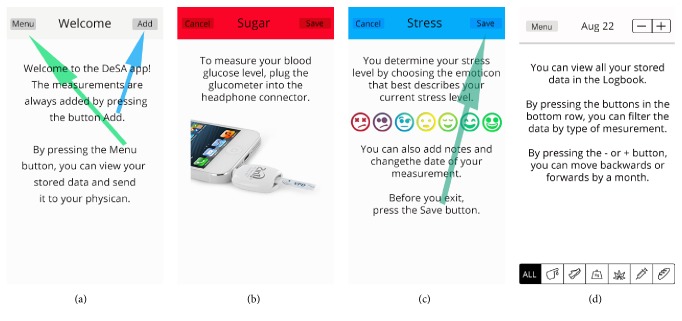
Added instructions for easier use.

After reviewing the questionnaire results, modifications were made in the app's appearance and functionalities. The first step was to add a welcome wizard, which helps a new user learn how to use the app (see Figure 4).

**Figure 5:**
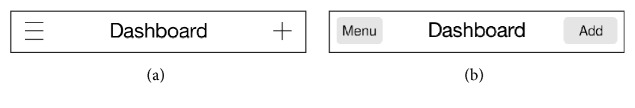
(a) The buttons of the original app on first launch; (b) the changed buttons.

The next step was to change the button symbols into text and enable the iOS accessibility functions which allow a user to enlarge text and, if needed, draw button shapes to make buttons more apparent (Figure 5(b)).

Additionally, error notifications and input checking (limiting values to meaningful numbers) were also added. Most users did not detect this problem, but it was observed by the moderator and could present a significant usability issue when used frequently.

Next, the text colour of the Add menu was changed to increase contrast, because some users had a problem with distinguishing black letters on the dark background.

### 4.2. Second Testing Phase

**Figure 6:**
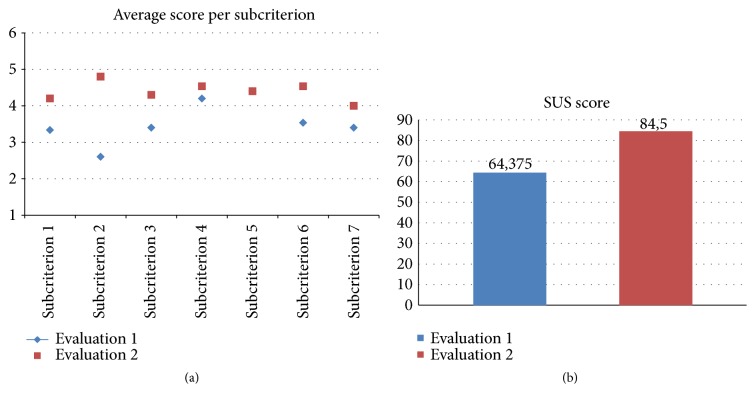
Questionnaire and SUS results.

The second testing phase was performed on a different set of users with the average age of 65. The same moderating technique was adopted and the users filled out both questionnaires (SUS and Table 1). The SUS results were slightly higher than the first evaluation with a score of 84.5 (see Figure 6(b)). All criteria rated better in the second evaluation, with the smallest difference in the criterion “presentation”, which was already the best graded category in the first test (with the average score of 4.5). The biggest difference in the modified app was noticed in the “comprehensibility” criterion, where the grade went from 3.1 to 4.4. The increase was also very apparent in the “usability” criterion, with an increase from 3.5 to 4.3 (see Figure 6(b)).

## 5. Discussion

Since the average age of the population in developed world is increasing, the number of chronic conditions is also on the rise. For all interventions involving mobile apps, certain aspects should be considered when trying to involve elderly users. Our own experience has shown that not involving elderly users in the design and development process can cause significant usability barriers in that demographic. This is especially true when such app is meant to be used by the same target population that also has the highest prevalence of the disease.

During the evaluations, two users stood out; one of them had no experience with touchscreen devices, used an old mobile phone for making calls only, had never used the Internet or sent any text messages, and had never used a computer. The other one was an advanced user that had used different sports apps in connection with body sensors to track his physical activity. Interestingly, they both had similar usability issues regarding the app. The only important difference was the advanced user's lack of fear of technology. A common problem with elderly users is their reluctance to press buttons due to the fear of breaking something. The advanced user clicked around the screen until he found what he was looking for, while the others spent a lot of time observing the screen and trying to determine the correct step.

The presented results are generic findings that would improve the usability for most users. But there are of course users that could still have problems using the app, especially users with limited technical proficiency. Such users would benefit from having assistance until they get comfortable with using new technology. A similar approach will be used in the project UNCAP where the technology training and familiarization have started long before the service is available to the users. This means the users will already be acquainted with different aspects of using mobile apps. Such training with caregivers also gives the developers valuable insight into what users want and need.

The most important points to keep in mind when designing an app for the elderly are thus the size, visibility, and comprehensibility of buttons and symbols. It can also be beneficial to combine symbols with text to increase clarity. Most modern devices (including those running iOS and Android) have built-in accessibility support that can be enabled in the app, in case users need to enlarge the text size, enable voice over, invert colours, and so forth. However, in case of iOS, the app needs to specifically support it, which is highly recommended.

Additionally, helpful tips and explanations must be available to the user. They can be in the form of a welcome wizard or as an additional button, which is always available to the user in case they need assistance. Age-related memory decline and not being very familiar with the technology can cause the user to become easily confused; therefore, it is very important to give them the possibility of looking for help. The general trend in this area is worrying, with device makers making unsubstantiated claims about their devices being simple to use, while this may increasingly not be the case anymore.

Next, colour contrast needs to be taken into account for users with poor eyesight; when using stronger background colours, the designer has to make sure the text remains legible. This problem is further exacerbated with modern high-resolution screens, which allow even thinner fonts with poorer legibility [28]. This goes hand in hand with the recent shifts from skeuomorphic to flat design mobile operating systems, as it has a significant impact on the overall usability. For example, earlier versions of iOS implemented a skeuomorphic design, styling the user interfaces with the specific goal to resemble real-world objects. This greatly aided the first-time users and educated them on how to use the applications by analogy. By ensuring the app has all the accessibility functions enabled, at least some of the issues attributed to flat design can be mitigated.

Another important aspect lies in the overlap of usability and data integrity. Handling exceptions and faults, as well as validating user input, is of crucial importance both for ease of use and for ensuring clean and valid data. This is especially important in mHealth apps, where medical decisions can be later made based on faulty data, having significant impact on the well-being of the patient.

We understand that the test users did not use all of the functionalities of the app, which could affect the overall score significantly. In [22], the authors came to the conclusion that the presence of documentation and analysis functions reduced the usability score significantly. Since the DeSA app was not compared to other apps, we could not determine whether an application with fewer functions would be preferable to the users; however, we do estimate that the larger number of features makes the app harder to use.

## 6. Conclusion

In this paper, we performed an evaluation of an existing diabetes app in two consecutive test trials with elderly users, using two different questionnaires in parallel. The overall results show, that applications developed for the general population are not necessarily suitable for elderly users, which can be a significant problem, especially if they address the issues of the elderly users specifically. We demonstrated that, with a limited amount of modifications, an existing app can be significantly improved to better suit elderly users. This could also be facilitated by creating different profiles to optimize the app for different accessibility groups (e.g., poor eyesight and limited dexterity). A user could simply select their profile and the app would be configured to their preferences. Such personalization features would of course have significantly larger impact and reach, if they were consistently implemented in all of the major mobile device operating systems. Making apps that would suit any and every user would be a very difficult if not impossible task. In particular, if one develops apps meant for a wide audience. The best one can currently do is focus on some characteristics that most users of the target group have in common and try to adapt the app to best suit their needs. Considering the number of older users that will need the help of mHealth apps in the future, it is increasingly important to focus our efforts on delivering beneficial solutions that will suit this demographic. This will hopefully help them take control of their disease and prolong their independence.

## Figures and Tables

**Table 1:** Questionnaire used in testing, adapted from [22].

Main criterion/subcriteria	Description of characteristics	Assessment criteria
Comprehensibility	*Use of understandable semantics* (i) Avoidance of foreign language and technical terms (ii) Use of generally intelligible symbols and terms (on buttons) (iii) If necessary, provision of additional explanations	5-point Likert scale (1 = does not apply at all; 5 = does fully apply)
	*Simple comprehensibility and interpretability of displayed images and depictions* (i) Self-explanatory images and depictions, understandable without further support and explanations	5-point Likert scale (1 = does not apply at all; 5 = does fully apply)
	*Simple, self-explanatory menu structures* (i) Easily understandable and internally consistent menu structures (ii) Avoidance of strong hierarchical menu structures and too many functionalities	5-point Likert scale (1 = does not apply at all; 5 = does fully apply)

Presentation (Image and Text)	*Sufficient colour contrast* (i) Clear, distinguishable colours for images and depictions or choice of colour-neutral depictions (ii) Avoidance of excessively glaring colours	5-point Likert scale (1 = does not apply at all; 5 = does fully apply)
	*Large size of operating elements* (i) Sufficient size of screen as well as input and output fields	5-point Likert scale (1 = does not apply at all; 5 = does fully apply)

Usability	*Intuitive usability* (i) Ability to use the application without prior knowledge (ii) Ease of learning (iii) Fast achievement of a first feeling of success	5-point Likert scale (1 = does not apply at all; 5 = does fully apply)
	*Simple recognition of click-sensitive areas* (i) Simple distinction between click-sensitive and non-click-sensitive areas, also without prior knowledge of the features of the touchscreen technology	5-point Likert scale (1 = does not apply at all; 5 = does fully apply)

General characteristics	*High fault tolerance/efficient fault management* (i) Reducing probability of erroneous data input by limiting choice to meaningful values (ii) Efficient proofreading mode and/or helpful user feedback, for example, in case of erroneous data input	5-point Likert scale (1 = does not apply at all; 5 = does fully apply)
